# Detection of Human Cytomegalovirus in Different Histopathological Types of Glioma in Iraqi Patients

**DOI:** 10.1155/2015/642652

**Published:** 2015-02-01

**Authors:** Haidar A. Shamran, Haider S. Kadhim, Aws R. Hussain, Abdulameer Kareem, Dennis D. Taub, Robert L. Price, Mitzi Nagarkatti, Prakash S. Nagarkatti, Udai P. Singh

**Affiliations:** ^1^Medical Research Unit, School of Medicine, University of Al-Nahrain, Baghdad 10066, Iraq; ^2^Microbiology Department, School of Medicine, University of Al-Nahrain, Baghdad 10066, Iraq; ^3^Department of Pathology, School of Medicine, University of Al-Qadisiya, Dewaniya 58011, Iraq; ^4^Department of Medical Science, School of Nursing, University of Al-Qadisiya, Dewaniya 58011, Iraq; ^5^Center for Translational Studies, Medical Services, VA Medical Center, Department of Veteran Affairs, Washington, DC 20422, USA; ^6^Department of Cell Biology and Anatomy, School of Medicine, University of South Carolina, SC 29208, USA; ^7^Department of Pathology, Microbiology, and Immunology, School of Medicine, University of South Carolina, SC 29208, USA

## Abstract

Human Cytomegalovirus (HCMV) is an endemic herpes virus that reemerges in cancer patients enhancing oncogenic potential. HCMV infection is associated with certain types of cancer morbidity such as glioblastomas. HCMV, like all other herpes viruses, has the ability to remain latent within the body of the host and can contribute in chronic inflammation. To determine the role of HCMV in glioma pathogenesis, paraffin-embedded blocks from glioma patients (*n* = 50) and from benign meningioma patients (*n* = 30) were obtained and evaluated by immunohistochemistry and polymerase chain reaction for the evidence of HCMV antigen expression and the presence of viral DNA. We detected HCMV antigen and DNA for IEI-72, pp65, and late antigen in 33/36, 28/36, and 26/36 in glioblastoma multiforme patients whereas 12/14, 10/14, and 9/14 in anaplastic astrocytoma patients, respectively. Furthermore, 84% of glioma patients were positive for immunoglobulin G (IgG) compared to 72.5% among control samples (*P* = 0.04). These data indicate the presence of the HCMV virus in a high percentage of glioma samples demonstrating distinct histopathological grades and support previous reports showing the presence of HCMV infection in glioma tissue. These studies demonstrate that detection of low-levels of latent viral infections may play an active role in glioma development and pathogenesis.

## 1. Introduction

Tumors of the central nervous system (CNS) represent about 2% of all cancers [1] and gliomas are the most common tumors of this system. In adults, gliomas account for almost 80% of primary malignant brain tumors [2]. Glioblastoma multiforme (GBM) is the most common type of glioma and is associated with a median survival of only 12–15 months [3]. In Iraq, CNS tumors are the fifth most common tumor in adults and the second most common in children [4]. However, these tumors are the most difficult to cure. For example, the total surgical removal of the affected part of the organ, which is used with other cancers, cannot be applied to cure brain tumors because each region of the brain has a vital function [5].

Human Cytomegalovirus (HCMV), a widespread beta-herpes virus, persistently infects over 70% of the population [6]. It has been shown that viral infections are responsible for approximately 15% of all cancers [7]. HMCV is associated with brain, breast, and colorectal cancer [8–10].

Geder et al. [11] demonstrated that genital isolates of HCMV could result in oncogenic transformation of human embryo lung fibroblasts. A group led by Cinatl Jr. et al. [12] demonstrated three oncomodulatory aspects of HCMV, which were the expression of oncogenes, transcriptional activation of growth factor, and interleukin synthesis. However, the role of this virus in brain tumor development has been a matter of debate for many years. A role for HCMV in brain tumors can be deduced from the association of anti-HCMV IgG and IgM Abs with the incidence of gliomas. This was confirmed by Amirian et al. [13], who found that anti-HCMV Abs level was associated with glioma risk, especially among IgM-positive individuals. In contrast, Sjöström et al. [14] did not find significant association between glioma or GBM and anti-HCMV IgG levels.

The presence of viral DNA or protein inside tumor versus nontumor cells can give more accurate and direct causal association than estimation of anti-HCMV antibodies. Cobbs et al. [15] shows that HCMV proteins and nucleic acids products are expressed in virtually all GBMs, but not in normal brain or other benign tumors suggest a possibility that HCMV may play an active role in glioma pathogenesis. Further, 92% of primary GBM tumors express viral immediate early protein, while 73% of them express late proteins [16]. In addition, HCMV in tumors and the peripheral blood of patients diagnosed with GBM shows a high percentage (>90%) of this tumor associated with HCMV nucleic acid and proteins, and 80% of patients had detectable HCMV DNA in their peripheral blood [17]. Recent studies have also reported the presence of HCMV proteins in 99% of brain tumor tissue sections isolated from GBM patients [18].

HCMV in glioma and other tumors is a major concern in Iraq and until now no published information has been available on the expression and association of this virus with these tumors. Studying HCMV expression and association with brain tumors might be beneficial in development of possible diagnostic and interventional strategies in these patients. To this end, in the present study, we investigated the expression represented by early (IEI-72), mid (pp65), and late HCMV antigens in different grades of GBM and anaplastic astrocytoma in Iraqi patients.

## 2. Materials and Methods

Patients from the Neurosurgery Hospital, Neuroscience Hospital, AL-Kadimiya Teaching Hospital, AL-Fallujah Hospital, and Private Nursing Home Hospital undergoing surgical resection of brain tumors from January 2012 to March 2013 were included in this study. Ethical approval to conduct the research was obtained from each hospital Institutional Review Board (IRB). Fifty paraffin-embedded brain tissues from the patients were used (mean age 31.85 ± 1.95 years, range 1–72 years). Personal data were collected through direct interview with the patient and by seeking his/her hospital record and medical records. Patient claims were followed as an alternative source of information when his/her previous medical reports were not available. Collected data included age, sex, smoking, and first relative family history of brain tumors. All brain tumors were graded by a histopathologist. Among the 50 specimens, 36 were GBMs and 14 were anaplastic astrocytoma. Thirty paraffin-embedded blocks from aged-matched benign meningioma patients were used as controls.

### 2.1. Immunohistochemistry (IHC)

All paraffin-embedded sections (6 *μ*m thick; for patients and control) were deparaffinized by heating slides in a vertical position in an oven for 1 hour followed by washing in xylene (20 min) and an ethanol series (100%, 95%, 75%, and 50% for 5 min each). Sections were blocked for endogenous peroxidase 3% H_2_O_2_ for 20 min (BioGenex, CA, USA). Antigen retrieval was performed using Citra Plus antigen retrieval solution (BioGenex, CA, USA) for 2 min at 97°C in a microwave. Protein blocks (EMD Millipore, IL, USA) were applied to the sections prior to application of primary antibodies (against the HCMV antigen IE1, pp65, and late antigen (1 : 100 for all antigens), EMD Millipore, IL, USA). Human lung with known HCMV infection was used as a positive control (Chemicon, Temecula, CA, USA) and negative control tumor sections that were not treated with an antibody stained were also included the experiments. All slides were incubated overnight at 4°C. The slides were then stained with secondary antibody biotinylated goat anti-mouse (1 : 17.5 IHC select HRP kit from EMD Millipore, IL, USA), peroxidase-labeled streptavidin (EMD Millipore, IL, USA), and 3,3′-diaminobenzidine (DAB) (Innovex Biosciences, USA). Hematoxylin was used as a counterstain. Human lung sections with known HCMV infection were used as positive controls for staining (Chemicon, Temecula, CA, USA). Negative controls consisted of tumor sections that were not treated with the primary antibody and/or secondary antibody. All samples were stained more than once and the result was highly reproducible.

### 2.2. Immunohistochemical Scoring

Immunostained sections were evaluated by two independent histopathologists, who were blinded to the patient information and disease severity. Cases with disagreement were discussed using a multiheaded microscope until agreement was achieved. For HCMV proteins (IE1-72, pp65, and late antigen), the brown staining of the nucleus with or without a diffuse pattern was considered positive. The intensity of protein expression was scored as reported by Rahbar et al. [18] by examining 1000 cells in 10 high power fields. According to the estimated percentage of positive cells four grades were identified: grade 1 <25%; grade 2 ≥25%–50%; grade 3 ≥51%–75%; grade 4 ≥76%.

### 2.3. DNA Extraction

A ready kit (DNeasy Blood and tissue kit/Qiagen, Valencia) was used for viral DNA extraction from tumor specimens according the manufacturer's instructions.

### 2.4. Polymerase Chain Reaction

In order to reduce contamination and prevent false positive results, preparation for PCR was carried out in a laboratory not previously used for handling HCMV. A nested PCR method was used to detect viral DNA. External primer sequences were the forward primer 5-CCGAAATACGCGTTTTGAGAT and the reverse primer 5-CCAAGCCAAAAACAGTATAGC, which amplified a 1300 bp of the promoter region of the immediate early (IE) gene. A ready master mix (Bioneer, Korea) was used for PCR reaction preparation. The PCR conditions were 95°C for 5 min, followed by 45 cycles of 94°C for 30 s, 56°C for 30 s, and 72°C for 1 min, with a final extension at 72°C for 10 min. Internal primer sequences were the forward primer 5-GGCGGAGTT (G/A) TTACGACATTT and the reverse primer 5-ATGCGGTTTTGGCAGTACAT, which amplified a 144 bp region within the 1300 bp amplicon generated by the external primer set. The PCR conditions were 95°C for 5 min, followed by 40 cycles of 94°C for 20 s, 56°C for 20 s, and 72°C for 20 s, with a final extension at 72°C for 5 min.

### 2.5. HCMV ELISA IgG

Anti-CMV IgG ELISA test kits (Biokit, Barcelona, Spain) were used for qualitative detection of serum anti-CMV IgG antibodies according to the manufacturer's manual.

### 2.6. Statistical Analysis

The Statistical Package for the Social Sciences (SPSS, version 14) was used for statistical analysis. The logistic regression test was used to test the association of HCMV immune positivity with incidence of gliomas. The chi-square test was used to compare immune positivity to Cytomegalovirus in different age classes and in evaluation of immunostaining. The correlation test was used to calculate *r*-values for HCMV protein expression. A *P* value < 0.05 was considered statistically significant.

## 3. Results

**Figure 1:**
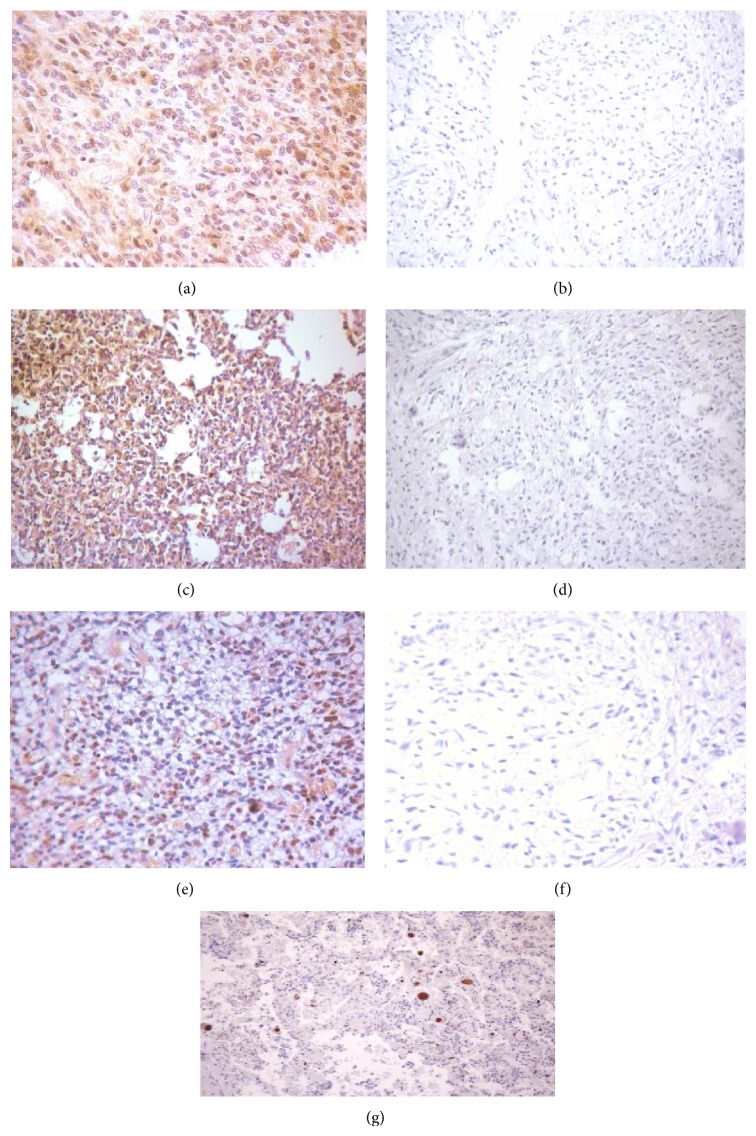
Immunohistochemical staining of HCMV in glioma. (a) Immunohistochemical stain of glioma section with IE1-72 protein. (b) Control without secondary antibody. (c) Immunohistochemical stains of glioma section with pp65 protein. (d) Control without secondary antibody. (e) Immunohistochemical staining of glioma with late antigen antibody. (f) Control without secondary antibody. (g) Lung known positive control stain for HCMV.

We performed IHC on paraffin sections from 50 gliomas that included 36 GBM and 14 anaplastic astrocytomas, using mAbs specific for HCMV proteins (IE1-72, pp65, and late antigen). Out of 36 GBM samples 33 (91.67%), 28 (77.78%) and 26 (72.22%) were positive for IE1-72, pp65, and late antigen, respectively (Table 1). We noticed no significant difference in the expression of these proteins between GBMs and anaplastic astrocytomas. Control tissue sections (meningioma biopsy specimens) and the surrounding brain area of malignant gliomas were devoid of immunoreactivity (Figure 1).

IE1-72 protein demonstrated stronger expression than other proteins, as 13 out of 33 positive GBM samples (39.39%) were grade 4 compared to 5 (17.58%) and 3 (11.54%) for pp65 and late antigen, respectively (*χ* ^2^ = 7.052, *P* = 0.029) (Table 1). Furthermore, IE1-72 had the lowest percentage of samples that were expressed as grade 1 (21.21%) compared to 25% for pp65 and 29.92% for late antigen. The correlation test revealed highly significant positive correlation between IE1-72, late antigen (*r* = 0.769, *P* < 0.001), and pp65 (*r* = 0.714, *P* < 0.001).

Among 14 anaplastic astrocytoma samples 12 (85.71%), 10 (71.42%), and 9 (64.82) were positive for IE1-72, pp65, and late antigen, respectively (Table 2). Out of 14 positive anaplastic astrocytoma samples, 6 (50%), 1 (10), and 1 (11.11%) were grade 4 for IE1-72, pp65, and late antigen, respectively, with a significant difference (*χ* ^2^ = 5.988, *P* = 0.05). Furthermore, IE1-72 protein had the lowest percentage of samples that were expressed as grade 1 (16.67%) compared to pp65 (20%) and late antigen (33.33%). A strong significant positive correlation appeared between pp65 and late antigen (*r* = 0.949, *P* < 0.001) and moderate correlation between IE1-72 and pp65 (*r* = 0.677, *P* = 0.032). No correlation between IE1-72 and late antigen (*r* = 0.567, *P* = 0.111) was found.

### 3.1. Polymerase Chain Reaction

**Figure 2:**
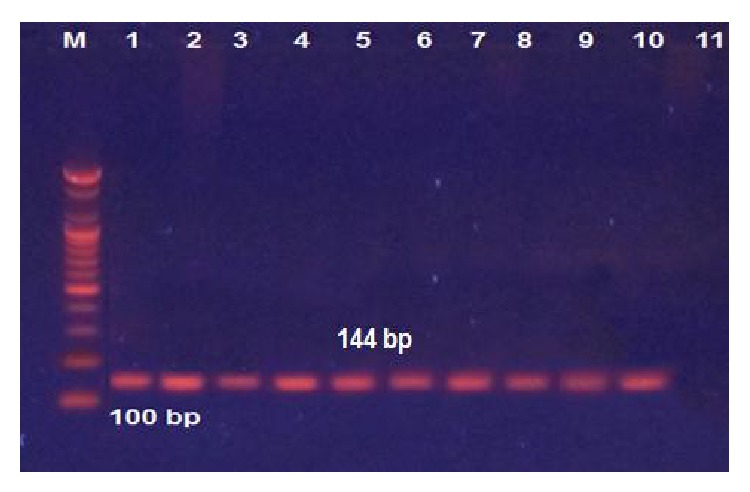
Detection of HCMV DNA in malignant glioma tissue (nested PCR products) visualized with UV light M: 1000 bp DNA marker. Lanes 1–10: viral DNA amplified with nested PCR. Lane 11: negative result. The size of the PCR product is 144 bp.

To determine the HCMV nucleic acids present, we selected cases that were positive for HCMV by IHC and extracted DNA from the original paraffin blocks for use in PCR reactions. A nested PCR approach was utilized to detect viral DNA. We detected HCMV nucleic acids in tumor cells, but not in control nontumor brain cells (Figure 2).

### 3.2. Seropositivity for Anti-HCMV IgG Antibodies

From 50 glioma patients, 42 (84%) were positive for anti-HCMV IgG antibodies, compared to 29/40 (72.5%) among the control group (Table 3). Logistic regression revealed a significant association between seropositivity for anti-HCMV IgG antibodies and glioma (OR = 2.779, 95% CI = 1.049–7.358, *P* = 0.04), which means that individuals who had a previous infection of HCMV are 2.779-fold more likely to get glioma compared with individuals with no such infection. Overall, there were no significant differences in the distribution of these antibodies among different age classes of glioma patients (Table 3). However, age classes of >50 and 31–40 years had the highest percentage (88.89%), while the lowest percentage (80%) was among the youngest age class (21–30 years). Among the control group, 21–30-year-old individuals had a higher seropositive percentage (91.67%) than other classes with significant differences (*P* = 0.037). Comparison of the age classes between glioma patients and controls revealed significant differences in the seropositivity, which was higher in glioma patients, in all classes except the 21–30 years class which was higher in the control group (91.67%) than that of glioma patients (80%).

## 4. Discussion

Studying viral proteins in malignant glioma patients may provide more details about the mechanism that these viruses use to modulate the cancer. To the best of our knowledge this is the first study from Iraqi patients, which showed HCMV protein and DNA in glioma specimens. The results of the current study indicate that 72.22% and 91.67% of GBM cases and 64.28% to 85.71% of anaplastic astrocytoma are positive for one or more HCMV proteins and nucleic acids. These results corroborate previous studies (16, 17, and 19) where HCMV had an obvious association with the occurrence of gliomas despite disparities in the percentage of tumor cells that gave positive results for the viral products.

In immunocytochemical studies, disparities in results are unavoidable because staining patterns vary by histology, cell type, and procedures followed during processing and staining of paraffin-embedded tissues. In this study, we followed all crucial steps during detection of HCMV infection in brain tumor tissue. We used brain tumor tissue of 6 *μ*m because thicker sections do not allow proper staining and thinner sections allow excessive nonspecific binding. This was corroborated by the study from Scheurer et al. [19]. Another crucial factor in the detection of HCMV in human gliomas is the dilution of antibodies used (1 : 100). Our results are similar to other studies done by Sabatier et al. [20] and Poltermann et al. [21], using a lower concentration of 1 : 200. The deparaffinization of slides may also play a role. In this study, we deparaffinized the slides by heating them in the microwave and treating with xylene for 20 minutes. Results show similarities with the study by Scheurer et al. [19] but disagree with another study [22] that used either microwave or steam heating for much longer periods, which may result in damage to IE1-72 epitopes.

The high prevalence of viral DNA and proteins in both GBM and astrocytoma and absence of these products in benign tumors and surrounding nontumor tissues indicate a vital role for this virus in carcinogenesis. However, tumor cells in general have a higher propensity to display false-positive immune-reactivity, due to either nonspecific binding to mAbs or higher levels of endogenous peroxidases that react with the detection substrate [17]. Furthermore, HCMV was found to use epidermal growth factor receptors (EGFR) as a cellular binding and incorporation site for its entry into the cell [23]. It has been shown that GMB cells uniformly amplified EGFR expression, while normal brain cells are largely negative [24]. Particularly with HCMV, which has lifetime latency, occurrence of the cancer and subsequent treatment induced immunosuppression cause reactivation of the latent virus and increase the chance for infection of the tumor cell as well as many other normal cells. Presence of the viral products inside the tumor cells is significant and important in the downregulation of immunogenicity of infected cells through multiple mechanisms, some of which are exhibited by the tumor cells themselves. These mechanisms involve inhibition of antigen presentation, downregulation of surface MHC expression, elaboration of transforming growth factor-beta (TGF-*β*) from infected cells, and secretion of viral IL-10 homologue (vIL-10) [25]. The special features acquired by the glioma cells might increase the tendency of HCMV to enter these cells, and this entry might confer protection for the HCMV-infected cells from immune-surveillance.

The nonsignificant differences in expression of the three HCMV proteins between GBM and anaplastic astrocytoma are an expected result. Cancer cells are exposed to almost the same conditions that facilitate the entrance of the virus into these cells. Of course not all tumor cells are simultaneously infected with the virus because the viral life cycle is markedly altered when it infects the cells. However, in one study, the authors reported 100% GBM positive cells for viral IE1-72 protein compared to 75% of cells being positive for the same protein in anaplastic astrocytoma [19].

The primary HCMV antigens, which induce cellular immune response to the virus, include immediate early protein (IE), virion envelope glycoprotein B (late antigen 55 kDa), and internal matrix protein (pp65) [26]. Accumulating evidence indicates that HCMV* IE1* products can interact with TP53 and Rb proteins (tumor suppressor proteins), and thereby it can induce cell cycle progression and block apoptosis. Indeed,* IE1 *expression in a glioma cell line significantly reduces* P53* expression [27]. Another study indicated that this protein interacts with the P13K/AKT pathway, one of the most important signaling pathways in glioma, and sustains activation of AKT signaling [28]. The viral protein pp65 (also called pUL83) is abundantly synthesized during lytic infection [29]. It is an important target of CD8^+^ T-cells in the course of immune response to HCMV infection [30]. Among the many functions it has, this protein is an immune-modulator and has been shown to block interferon activity [31] and downregulate the interferon-response gene primarily through modulation of interferon response factor-3, IRF-3 [32].

A major late antigen of HCMV is glycoprotein B (gB). It is a type 1 trans-membrane protein that exists as a dimer on the surface of the viral envelope [33]. This antigen is implicated in the virus entry, cell-to-cell spread [34], and fusion of the infected cells [35]. There is no evidence from available reports that this antigen has any role in cell transformation, and the positive result for the presence of the antigen inside the tumor cells, but not the nontumor ones, may be related to the features of tumor cells that facilitate the entrance of HCMV compared to the normal cells. One of the most important features of IE1-72 protein is early production of this protein in the nucleus of the infected cell. It is because of this property that IE1-72 has stronger expression than pp65, which in turn has stronger expression than the late antigen. The last gene is known to encode for an envelope glycoprotein, and in many instances it scatters on the cell membrane of the infected cell and cannot be easily detected. These results are in part in accordance with a previous study by Mitchell et al. [17], who stated that pp65 reactivity is less ubiquitous than IE1-72 in tumor cells.

A significant correlation between the level of pp65 and gB antigens in the MRC-5 cell line experimentally infected with HCMV has been reported by Jun et al. [36]. It is not unusual to find such correlation among viral proteins as the infection occurs in defined sequences chronologically accompanied by expression of immediate early, early, and late viral antigens. Hence, HCMV proteins are expected to follow the pattern of elevation and decline shown by immediate early proteins, but to a lesser degree.

Further support for the role of HCMV in the development of this tumor results from the use of anti-HCMV drugs. Some studies have shown that valganciclovir significantly reduced the growth of HCMV-positive glioma tumors [37]. Recently Baryawno et al. [38] demonstrated an increase in overall survival of glioblastoma patients receiving valganciclovir compared with those patients who did not receive such treatment.

The results from the present study show the high percentage of HCMV virus expression in different histopathological grades of glioma patients, which clearly suggest its role in glioma pathogenesis. The detection of low-level latent HCMV infections might suggest a role in glioma pathogenesis. However, a more detailed study of HCMV infection in glioma patients is required for a definitive conclusion.

## Figures and Tables

**Table 1:** The percentage and grading of HCMV protein in GBM.

HCMV proteins Number = 36	Positivity Number (%)	Grading of positive sections			
		Grade 1 Number (%)	Grade 2 Number (%)	Grade 3 Number (%)	Grade 4 Number (%)
IE1-72	33 (91.67)	7 (21.21%)	6 (18.18)	7 (21.21)	13 (39.39)
pp65	28 (77.78)	7 (25)	9 (32.14)	7 (25)	5 (17.58)
Late antigen	26 (72.22)	7 (26.92)	10 (38.46)	8 (30.77)	3 (11.54)

Percentage changes and grading in early, mid, and late antigen HCMV protein in glioblastoma multiforme patients. The grade number and percentage (%) were calculated in a blinded fashion. The number and % of positive patients are indicated for each antigen.

**Table 2:** The percentage and grading of HCMV protein in anaplastic astrocytoma.

HCMV proteins Number = 14	Positivity Number (%)	Grading of positive sections			
		Grade 1 Number (%)	Grade 2 Number (%)	Grade 3 Number (%)	Grade 4 Number (%)
IE1-72	12 (85.71)	2 (16.67)	2 (16.67)	2 (16.67)	6 (50)
pp65	10 (71.42)	2 (20)	5 (50)	2 (20)	1 (10)
Late antigen	9 (64.28)	3 (33.33)	3 (33.33)	2 (22.22)	1 (11.11)

Percentage changes and grading in early, mid, and late antigen HCMV protein in anaplastic astrocytoma patients. The grade number and percentage (%) were calculated in a blinded fashion. The number and % of positive patients are indicated for each antigen.

**Table 3:** The distribution of seropositivity for anti-HCMV IgG antibodies among different age classes in glioma patients and control group.

Age class (years)	Glioma patients					Control				
	Number	Seropositive		Seronegative		Number	Seropositive		Seronegative	
		Number	%	Number	%		Number	%	Number	%
≤20	11	9	81.81	2	18.18	9	6	66.67	3	33.33
21–30	10	8	80	2	20	12	11	91.67	1	8.33
31–40	9	8	88.89	1	11.11	10	7	70	3	30
41–50	11	9	81.81	2	18.18	7	4	57.14	3	42.86
>50	9	8	88.89	1	11.11	2	1	50	1	50
